# Genome-wide analysis of *Burkholderia* for the management of antimicrobial-resistant in cystic fibrosis patients

**DOI:** 10.1186/s13568-025-01926-1

**Published:** 2025-08-26

**Authors:** Vijayavidhya Magizhvannan, Shanthi Veerappapillai

**Affiliations:** Department of Biotechnology, School of Bio Sciences and Technology, Vellore Institute of Technology, Vellore, Tamil Nadu India

**Keywords:** Antimicrobial resistance, *Burkholderia cepacia* complex, Protein–protein interaction, Cytoscape, Hub gene, Functional enrichment

## Abstract

*Burkholderia* is a significant pathogen that causes disease burden across the globe. In particular, *Burkholderia cenocepacia* and *Burkholderia multivorans* are the predominant isolates that infect people with cystic fibrosis (CF) and cause hospital-acquired infections. Understanding antimicrobial resistance and virulent factors among these species is of great significance for addressing this growing resistance burden. Initially, we retrieved 75 complete genome sequences of *B. cenocepacia* and *B. multivorans* from NCBI database and analysed them for antimicrobial resistance (AMR) and virulent factors. This yielded 368 antimicrobial resistance genes and 202 virulent factors after removing the duplicates. Further, a comprehensive interaction network was constructed using STRING, which was visualized and analysed using Cytoscape. Through cytoHubba and MCODE analysis, eight key hub genes *FliF, FliG, FliM, FliS, FlgB, FlgC, FlgD* and *FlgK* were identified. Additionally, a non-homology analysis was conducted to ensure that the key nodes do not exhibit similarity with the human genome and gut microbiota. Functional enrichment analysis revealed their significant role in the flagellar assembly pathway, particularly in bacterial motility, colonization and biofilm formation. Notably, seven hub genes were enriched in bacterial-type flagellum-dependent cell motility pathway and cellular localization. It is worth noting that 17,967 phytochemicals were exploited to identify the potent hit compounds against each of the identified hub genes. Interestingly, the hit molecules were found to form several key interactions with the targets, indicating their potential as promising therapeutic agents for combating AMR. Overall, the identified hub genes and their potent inhibitors present compelling targets for novel antimicrobial therapies in CF, underscoring the need for future experimental validation.

## Introduction

Anti-microbial resistance (AMR) is an emerging world-wide health concern that poses a significant threat to effective disease management with around 1.2 million deaths per year (Tang et al. 2023; Endale et al. 2023). Among the wide spectrum of multidrug resistant organisms, the genus *Burkholderia,* a gram-negative and opportunistic bacterial species has garnered considerable attention due to its high adaptability, resistance and ability to cause severe infections (Ahmed et al. 2024). *Burkholderia* encompasses over 70 species, including notable opportunistic pathogens such as *Burkholderia cepacia* complex (BCC) and *Burkholderia pseudomallei* (BPM). Remarkably, these pathogens are responsible for nosocomial infections, particularly in immunocompromised individuals and those with chronic lung diseases (Badten and Torres 2024). In particular, the pathogenicity of these bacteria is chiefly ascribed to their virulence factors, encompassing biofilm formation, efflux pumps, immune evasion strategies and the existence of particular virulence genes that enhance their capacity to induce disease.

Cystic fibrosis (CF) is a genetic disorder that is caused by a mutation in the Cystic Fibrosis Transmembrane Conductance Regulator gene (CFTR), resulting in the dysfunction of the chloride channel (Shmarina et al. 2024; Akram et al. 2024). This dysfunction leads to an increase in the mucus viscosity in the lower respiratory tract and a compromised mucociliary clearance, fostering an environment conducive to the colonization of various opportunistic pathogens (Ribeiro et al. 2023; Thornton and Parkins 2023). Notably, *B. cenocepacia* and *B. multivorans*, which are known to be the most virulent species of this complex, account for 85–97% of lung infections caused by BCC in this population globally (Santana and Jiménez 2024). In individuals with cystic fibrosis, BCC with the predominant isolates *B. cenocepacia* and *B. multivorans* can induce severe pulmonary infections that result in expedited lung deterioration, potentially complicated by necrotizing pneumonia characterized by elevated fevers, leucocytosis and bacteraemia, frequently leading to fatal consequences. In recent years, *Burkholderia* indicates a concerning prevalence of resistance to antibiotics, including cephalosporins and carbapenems (Saeed et al. 2024). Moreover, reports from the literature highlight that more than 50% of clinical isolates of BCC demonstrate resistance against multiple antibiotics, leading to intensified morbidity and mortality. Based on these frames of reference, addressing the AMR epidemic associated with *Burkholderia* species necessitates a comprehensive strategy that emphasizes the development of effective therapeutic approaches.

In recent decades, the protein–protein interaction network has gained immense importance as a way to identify potential biomarkers (Dorella et al. 2013). A multitude of studies underscore the importance of identifying biomarkers using protein–protein interaction (PPI) network. For instance, Pinto et al. (2023) utilized functional network analysis on *Stenotrophomonas maltophila* and identified 8 hub genes linked to antibiotic resistance and virulence pathways that may serve as therapeutic targets. Similarly, Gollapalli et al. 2021 examined the PPI network of essential genes in *Helicobacter pylori* to identify potential therapeutic targets. The study reported *guaA*, *dnaK* and *recA* as critical hub genes for bacterial survival and could be potential drug targets. In another study, Naha et al. (2020) constructed a gene interaction network and identified *Enterococcus faecalis* V585 as multi-drug resistant (MDR). It identified key genes involved in resistance that could be potential therapeutic targets to combat MDR. Notably, Wisal et al. (2024) adopted a hierarchical subtractive genomics pipeline to identify and virtually screen druggable targets in *Staphylococcus aureus,* incorporating molecular docking and Molecular Mechanics-Generalized Born Surface Area (MM-GBSA) analysis. These computational pipelines represent a scalable framework for tackling AMR. Although, these methods have proven effective against other pathogens, there remains a notable absence of comprehensive studies focused on *B. cenocepacia* and *B. multivorans.* Therefore, we employed an extensive genome-wide analysis integrating PPI network approach to identify the key genes associated with resistance. These candidates were virtually screened and evaluated to identify potential therapies for resistant *Burkholderia* infections in CF patients.

## Methodology

### Retrieval of the whole genome and acquisition of antimicrobial, virulent factors

The query for *B. cenocepacia* and *B. multivorans* was retrieved from the National Centre for Biotechnology Information (NCBI) database (https://www.ncbi.nlm.nih.gov/datasets/genome/?taxon=95486) Accessed November 12, 2024, yielding 1486 entries (de Aguiar et al. 2016). A total of 1411 incomplete genome sequences were discarded by in-built parameters. This preprocessing yielded 75 complete genome sequences. In the present study, the AMR and virulent genes were retrieved using the Resistant Gene Identifier tool 6.0.3 (RGI) of the Comprehensive Antibiotic Resistance Database (CARD, https://card.mcmaster.ca/analyze/rgi, Accessed November 22, 2024) (Rao et al. 2022; Alcock et al. 2023) and Virulence Factor Database (VFDB, https://www.mgc.ac.cn/VFs/, Accessed November 27, 2024 (Liu et al. 2021) respectively. The default parameters were set to obtain annotations based on complete gene match criteria for the identification of antibiotic resistance genes and virulent factors.

### Gene network construction and Identification of hub genes through cluster analysis

To identify the interacting genes in the network, the Search Tool for the Retrieval of Interacting Genes (STRING_v.11.5, https://string-db.org/) database was used (Szklarczyk et al. 2022). The confidence level was set to be 0.9 to identify highly interconnected genes (Hassan et al. 2016). The network type chosen was full STRING network where the edges indicate both functional and physical protein associations. Indeed, highly interacting genes play a crucial role in the antimicrobial activity of the species. The PPI network was visualized and further analysed using Cytoscape_v3.10.2. The disconnected nodes were excluded to enhance the interpretability of the network topology.

The PPI network was further analysed to gain insights into the relationship between AMR and virulence factors. Using cytoHubba, a plug-in in Cytoscape, the PPI network was screened for its hub genes (Chin et al. 2014). The PPI network ranked the top 10 genes through the application of each of the 12 in-built algorithms available in cytoHubba. The genes that were identified as hub genes in the PPI network overlapped in more than 6 algorithms and were assumed to play a critical role in AMR and virulence. Later, we utilized Molecular Complex Detection (MCODE), a plug-in in Cytoscape, to identify highly linked with clusters within the PPI network.

### Topological analysis

Topological analysis offers valuable insights into the structure and dynamics of the PPI network by identifying hub proteins and key central nodes. The intricacy of the complicated network was then assessed using NetworkAnalyzer, a plug-in in Cytoscape, according to topological metrics. The nodes in the network were evaluated in this study using five fundamental properties: degree, betweenness centrality, closeness centrality, clustering coefficient and average shortest path length coefficient.

### Non-homology analysis and essentiality analysis

To assure drug safety against off-target effects, the chosen target genes neither exhibit homology with host or human genes nor with the human gut microbiome (Wisal et al. 2024). Each target sequence of gene was compared to the complete human genome (taxid: 9606) and the essential gut microbes with the bacterial non-redundant database utilizing NCBI-BLASTp (Hassan et al. 2022; Basharat et al. 2021). An e-value of < 0.0001 and a minimum identity of ≥ 25% were established as the threshold for exclusion. To identify indispensable targets for bacterial survival, the non-homologous genes were compared to the Database of Essential Genes (DEG) using BLASTp (e-value = 0.001) (Basharat et al. 2022).

### Functional enrichment analysis

Functional enrichment analysis was conducted using the web-based tool ShinyGO (v0.80; http://bioinformatics.sdstate.edu/go/) (Ge et al. 2019). The hub genes were examined for the prevalence of Gene Ontology (GO) terms in Biological Processes (BP), Cellular Components (CC) and Molecular Functions (MF). Additionally, Kyoto Encyclopedia of Genes and Genomes (KEGG) (https://www.genome.jp/kegg/pathway.html) pathway enrichment was performed to identify overrepresented metabolic and signalling pathways. To ensure statistical rigor, enrichment significance was evaluated using the hypergeometric distribution. The *p* values were adjusted for multiple testing using the Benjamini–Hochberg False Discovery Rate (FDR) method. Pathways with an FDR-adjusted *p* value < 0.05 were considered statistically significant. This threshold enhances the reliability of gene selection by reducing false positives.

### Dataset retrieval

The AlphaFold Protein Structure Database was used to retrieve the 3D structures of *FliM* (AF-A0A1V6L0G0-F1-v4), *FliG* (AF-A0A109EK38-F1-v4), *FliF* (AF-A0A3Q9FAS5-F1-v4), *FlgB* (AF-A0A427NS22-F1-v4), *FlgC* (AF-A0A3R9CV50-F1-v4), *FlgD* (AF-A0A3S9NA56-F1-v4) and *FlgK* (AF-A0A427NS23-F1-v4) (https://www.alphafold.ebi.ac.uk/). Phytochemical compounds were retrieved from the IMPPAT (Indian Medicinal Plants, Phytochemistry And Therapeutics) database. Notably, phytochemicals are bioactive plant derived compounds known for their potent antimicrobial properties, making them promising candidates against multidrug-resistant pathogens (Abass et al. 2024). Compounds were filtered using drug-likeness with zero violation towards Lipinski’s RO5, Ghosh, Pfizer 3/75, Veber and Egan rules. Only compounds with zero violations and a weighted Quantitative Estimate of Drug-likeness (QEDw) > 0.7 were selected for screening.

### Molecular docking

Prior to the docking studies, the active site of the proteins was predicted using SiteMap module of Schrödinger suite. The top ranked site for each protein based on the SiteScore and DScore metrics, was selected for grid generation. Following site identification, a receptor grid was generated using Receptor Grid Generation wizard in Schrödinger’s Maestro interface (Alyahyawy et al. 2025). This grid thrived as the interaction site for subsequent molecular docking. Later, the ligand dataset was pre-processed using LigPrep with appropriate protonation states and OPLS3e force field minimization. Further, these compounds were docked against the hub proteins using Extra Precision (XP) mode of Glide.

### Binding free energy estimation

The binding free energies of the protein–ligand complexes were evaluated using the Prime module within the Schrödinger suite as a post docking validation. Previous studies have demonstrated that the binding energy predictions obtained through the Prime MM-GBSA method shows strong agreement with experimental results (Tilwani et al. 2023; Dasmahapatra et al. 2022). Specifically, the MM-GBSA calculations implemented in the Prime module incorporate the VSGB polar solvation model and OPLS force field, van der Waals interactions and solvent accessible surface area. In this study, the pose viewer files generated from Glide XP docking were used as the input for MM-GBSA to compute the binding free energy (ΔG_bind_) of each complex using the following equation:

$$ \begin{aligned} &\Delta {\text{G}}_{{{\text{bind}}}} = {\text{ E}}_{{{\text{complex}}({\text{minimized}})}} \\ &\quad - \left( {{\text{E}}_{{{\text{ligand}}({\text{minimized}})}} + {\text{ E}}_{{{\text{receptor}}({\text{minimized}})}} } \right) \end{aligned}$$

where E_complex(minimized)_ denotes the minimized energy of the complex; E_ligand(minimized)_ indicates the energy of the ligand after its separation from the complex; E_receptor(minimized_) represents the energy of the receptor after being separated from the ligand.

**Fig. 1:**
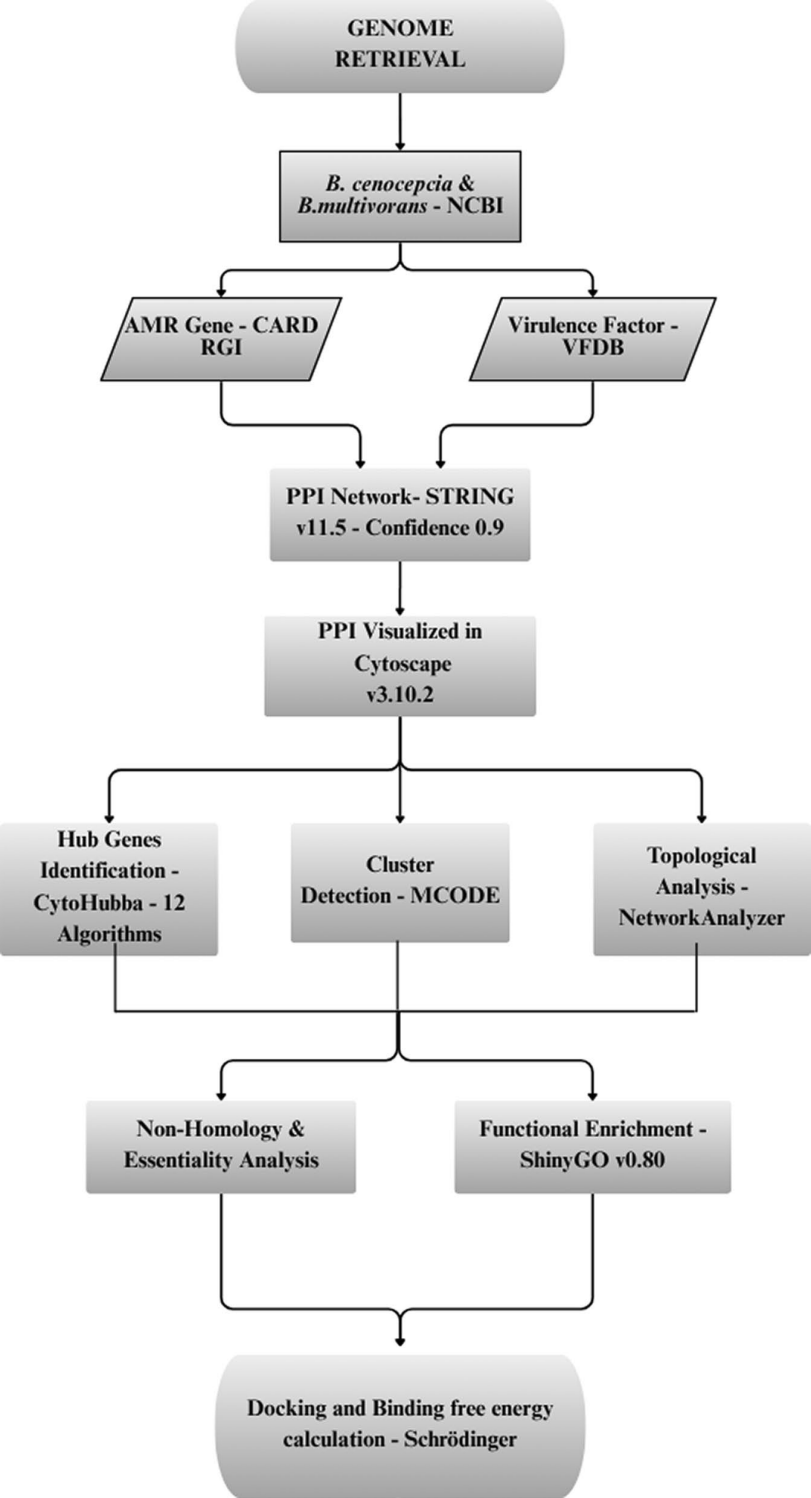
Schematic representation of the *in-silico* pipeline for hub gene identification and validation

A schematic representation of the entire methodology is illustrated in Fig. 1.

## Results

### Identification of antimicrobial resistance and virulence gene

The study exploited 75 whole genome sequences corresponding to *B. cenocepacia* and *B. multivorans* from the NCBI database to retrieve AMR genes and virulent factors (VF). The analysis yielded a total of 53,471 AMR genes and 10,979 VF were obtained from CARD and VFDB respectively. We carefully removed the duplicates using a thorough manual curation approach. In the end, a total of 368 AMR genes and 202 VFs were considered for further analysis.

### Identification of hub genes through protein–protein interaction and cluster analysis

In the present study, a PPI network was constructed using the STRING database with a confidence level of 0.9 and visualized using Cytoscape (Lv et al. 2024). The obtained PPI network consisted of 57 nodes interconnected by 554 edges after removing all the loosely bounded connections. Subsequently, the hub genes were identified using cytoHubba. Table 1 exemplifies the top 10 genes ranked by 12 algorithms of cytoHubba. We identified *FliM, FliG, FliF, FliS, FlgB, FlgC, FlgD* and *FlgK* genes as crucial genes that interact with most of the 6 algorithms, highlighting their importance (Rivkind et al. 2020). These nodes are vital for the cell's survival and are deemed essential genes of the pathogen.

**Table 1:** Top 10 genes ranked by cytoHubba

S. No	Betweenness	Bottleneck	Closeness	Clustering Coefficient	Degree	DMNC	EcCentricity	EPC	MCC	MNC	Radiality	Stress
1.	*CheA*	*MotB*	*FliM*	*CheY*	*FliM*	*FliN*	*FlhA*	*FlgD*	*FlgC*	*FliM*	*FliM*	*MotB*
2.	*MotB*	*CheA*	*FliG*	*CheD*	*FlgC*	*FlgF*	*MotA*	*FliG*	*FlgD*	*FlgC*	*FliG*	*CheA*
3.	*FliM*	*CheW*	*FlgC*	*FlhG*	*FlgD*	*FliI*	*MotB*	*FlgG*	*FlgB*	*FlgD*	*FliS*	*FliA*
4.	*FliA*	*MdtA*	*FlgD*	*PilD*	*FlgB*	*FliP*	*CheA*	*FliM*	*FlgG*	*FlgB*	*FlgK*	*FliM*
5.	*FliG*	*FliD*	*FlgB*	*PilC*	*FliG*	*FliH*	*FliM*	*FlgK*	*FliF*	*FliG*	*FlgC*	*FliG*
6.	*FliS*	*FliA*	*FliS*	*FliR*	*FlgG*	*FlgH*	*FliG*	*FliE*	*FliE*	*FlgG*	*FlgD*	*FliS*
7.	*FlgK*	*CheR*	*FlgK*	*FlgM*	*FliS*	*FliQ*	*FliS*	*FlgC*	*FliG*	*FliS*	*FlgB*	*FlgK*
8.	*FlhA*	*PilB*	*FlgG*	*FlgF*	*FlgK*	*FlgI*	*FlgK*	*FliL*	*FliM*	*FlgK*	*FlgG*	*FlhA*
9.	*CheZ*	*FlgB*	*FliF*	*FliI*	*FliF*	*FliF*	*FliK*	*FlgB*	*FlgH*	*FliF*	*FliF*	*FlgC*
10.	*MotA*	*FlgJ*	*FliE*	*FliQ*	*FliE*	*FliE*	*FliI*	*FliF*	*FliN*	*FliE*	*FliE*	*FlgD*

**Fig. 2:**
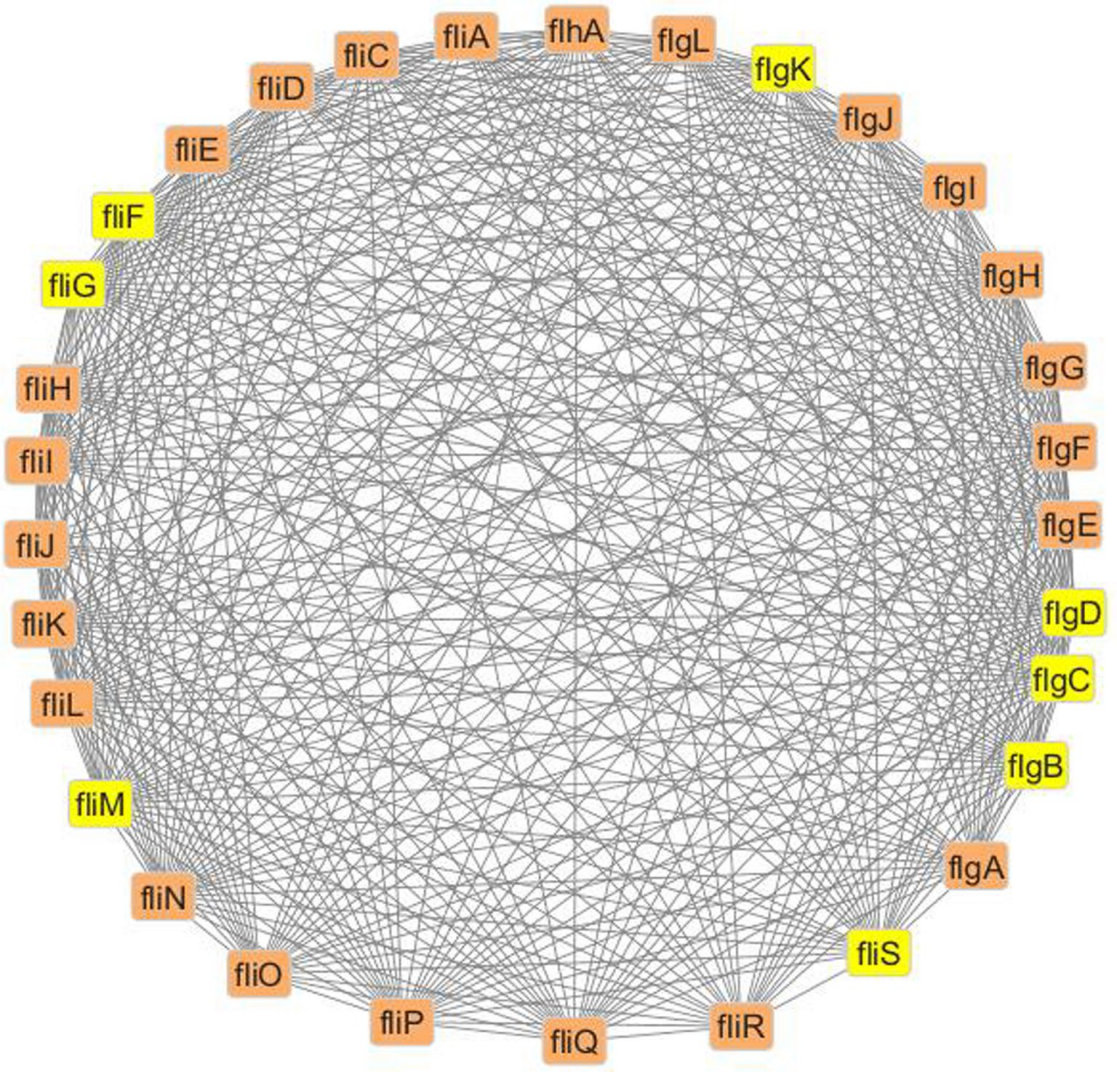
Cluster analysis of antimicrobial resistance (AMR) and virulence factor (VF) genes in *Burkholderia* species using MCODE. The highlighted nodes (Yellow) represent those with the highest levels of interconnectivity

Further, MCODE analysis was carried out which resulted in a highly interconnected cluster with 31 nodes, having 514 edges containing 8 hub nodes (Fig. 2). Thus, based on cytoHubba and MCODE analysis, genes such as *FliM, FliG, FliF, FliS, FlgB, FlgC, FlgD* and *FlgK* were selected for further analysis.

The chosen target genes must not have any similarity with host/human genes or the human gut flora to assure medication safety against the danger of off-targeting. Thus, NCBI-BLASTp was used to screen the non-homology against both the human genome (taxid: 9606) and a representative panel of essential gut microbiota. None of the targets showed any significant hits. This signifies non-homology of the identified hub genes with the human genome or gut microbiota. Further, to confirm essentiality, the non-homologous genes were subjected to BLASTp against the DEG database (Sohail et al. 2025). Targets shown significant similarity to essential bacterial genes, reinforcing their potential as viable therapeutic targets. This supports the safety and therapeutic relevance of the identified hub genes for drug development.

### Topology of PPI network

**Fig. 3:**
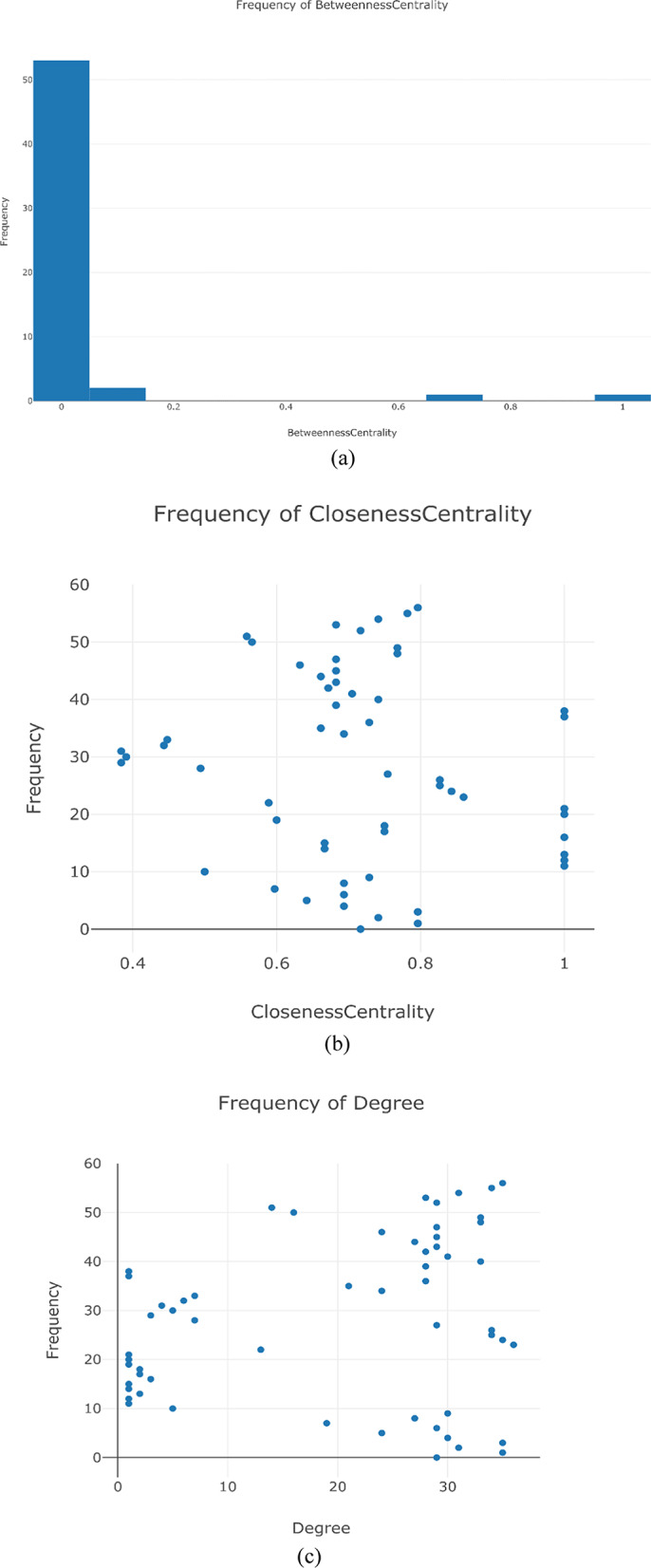
Topological analysis of a protein–protein interaction network for essential genes **a** Betweenness centrality distribution, **b** closeness centrality distribution, **c** degree distribution

The connections and interactions between genes involved in various cellular and metabolic processes are assessed by topological parameter analysis. Figure 3a–c show the betweenness centrality, closeness centrality and degree distribution of the nodes in the network. AMR genes and their direct interactors have substantial relationships, as evidenced by lower average shortest path length, greater degree and clustering coefficient **(**Table 2**)** (Naha et al. 2020). For instance, the average shortest path length indicates the distance from an individual node to every other node in the network. Consequently, a lower value signifies a more critical gene within the network. The analysis indicates that the shortest path length ranges from 1.162 for *FliM* to 1.302 for *FliF*. This suggests that *FliM* is the most centrally located gene within the network. Betweenness centrality measures the frequency with which a node acts as a connector on the shortest paths linking other nodes. Among the genes analysed, *FliM* shows the highest betweenness centrality (0.042), underscoring its crucial function in maintaining the network's connectivity. Further, closeness centrality measures how effectively a node can connect to every other node in a network. The results highlight that *FliM* shows the greatest closeness centrality (0.86), highlighting its ability for swift and effective connectivity to other nodes. The clustering coefficient indicates how interconnected a node's neighbors are with one another. *FliF* shows the highest clustering coefficient (0.852), reflecting a significant degree of interconnectivity among its neighboring nodes, which together create a cohesive subgroup. The degree quantifies the number of direct links related to a node within the network. An advanced degree indicates greater impact and opportunities for engagement within the framework. *FliM* holds a notable degree of 36, showcasing its prominence as a significant centre with broad interconnections. This analysis delineates the distinct functions of genes within the network *FliM* emerges as the most pivotal and substantial gene, showcasing the highest standings in average shortest path length, betweenness centrality, closeness centrality and degree. Similar strategies on network topology have been employed in other pathogens. Comparable studies in *Enterococcus faecalis* and *Clostridium difficile* have demonstrated similar trends, where topologically central genes (*pbpC*, *murE* and *rpoB*) which play pivotal roles in antibiotic resistance were identified (Naha et al. 2020; Anusha et al. 2023). These findings highlight the relevance of topological analysis in identifying promising therapeutic targets across diverse pathogens**.**

**Table 2:** List of hub genes with average shortest path length, betweenness centrality, closeness centrality, clustering coefficient and degree analysed using network analyser

S. No	Gene	Average shortest path length	Betweenness centrality	Closeness centrality	Clustering coefficient	Degree
1.	*FliM*	1.162	0.042	0.86	0.746	36
2.	*FliG*	1.186	0.037	0.843	0.773	35
3.	*FliS*	1.209	0.031	0.826	0.766	34
4.	*FlgK*	1.209	0.030	0.826	0.778	34
5.	*FlgB*	1.255	0.010	0.796	0.801	35
6.	*FlgD*	1.255	0.010	0.796	0.801	35
7.	*FlgC*	1.255	0.010	0.796	0.801	35
8.	*FliF*	1.302	0.006	0.767	0.852	33

### Pathway enrichment analysis

**Fig. 4:**
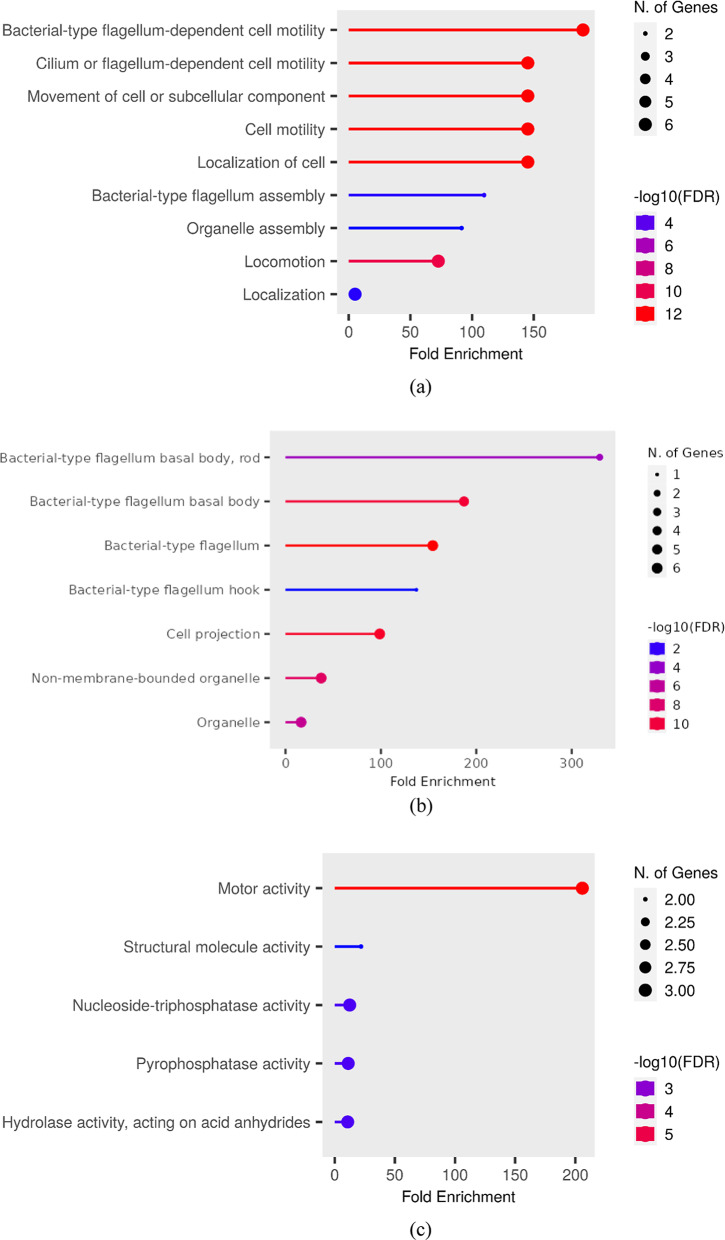
Functional enrichment analysis **a** Biological process, **b** cellular components, **c** molecular function

In order to acquire a better understanding of the biological functions for the identified hub genes, we accomplished GO and KEGG pathway (Khan et al. 2023; Ali et al. 2013) enrichment analyses. Table 3 and Fig. 4a results suggest the majority of the hub genes in the BP module were primarily enriched in bacterial-type flagellum-dependent cell motility, cilium or flagellum-dependent cell motility, movement of cell or subcellular component, cell motility, localization of cells and locomotion. Table 4 and Fig. 4b indicates that CC terms of hub genes are associated with bacterial flagellum, cell projection and non-membrane bound organelle. Table 5 and Fig. 4c indicates the MF of the genes was involved in motor activity, nucleoside-triphosphatase activity, pyrophosphatase activity, hydrolase activity and acting on acid anhydrides.

**Table 3:** Enriched biological processes associated with hub genes based on GO analysis, indicating key functional roles

S. No	Number of hub genes involved	Total number of genes involved in the pathway	Fold enrichment	Pathway	Hub genes
1.	6	26	189.989	Bacterial-type flagellum-dependent cell motility	*FlgC, FlgB, FliS, FliF, FliG, FliM*
2.	6	34	145.2857	Cilium or flagellum-dependent cell motility	*FlgC, FlgD, FliS, FliF, FliG, FliM*
3.	6	34	145.2857	Movement of cell or subcellular component	*FlgC, FlgB, FliS, FliF, FliG, FliM*
4.	6	34	145.2857	Cell motility	*FlgC, FlgB, FliS, FliF, FliG, FliM*
5.	6	34	145.2857	Localization of cell	*FlgC, FlgB, FliS, FliF, FliG, FliM*
6.	2	15	109.7714	Bacterial-type flagellum assembly	*FlgK, FliS*
7.	2	18	91.47619	Organelle assembly	*FlgK, FliS*
8.	6	68	72.64286	Locomotion	*FlgC, FlgB, FliS, FliF, FliG, FliM*
9.	6	952	5.188776	Localization	*FlgC, FlgB, FliS, FliF, FliG, FliM*

**Table 4:** GO-enriched cellular components related to hub genes, highlighting their subcellular localization

S. No	Number of hub genes involved	Total number of genes involved in the pathway	Fold Enrichment	Pathway	Genes
1.	2	5	329.3143	Bacterial-type flagellum basal body, rod	*FlgC, FlgB,*
2.	5	22	187.1104	Bacterial-type flagellum basal body	*FlgC, FlgB, FliF, FliG, FliM*
3.	6	32	154.3661	Bacterial-type flagellum	*FlgK, FlgC, FlgB, FliF, FliG, FliM*
4.	1	6	137.2143	Bacterial-type flagellum hook	*FlgK*
5.	6	50	98.79429	Cell projection	*FlgK, FlgC, FlgB, FliF, FliG, FliM*
6.	6	132	37.42208	Non-membrane-bounded organelle	*FlgK, FlgC, FlgB, FliF, FliG, FliM*
7.	6	301	16.41101	Organelle	*FlgK, FlgC, FlgB, FliF, FliG, FliM*

**Table 5:** Enriched molecular functions of hub genes derived from GO analysis, reflecting their functional activities

S. No	Number of hub genes involved	Total number of genes involved in the pathway	Fold enrichment	Pathway	Genes
1.	3	12	205.8214	Motor activity	*FliF, FliG, FliM*
2.	2	75	21.95429	Structural molecule activity	*FlgK, FliS*
3.	3	197	12.53735	Nucleoside-triphosphatase activity	*FliF, FliG, FliM*
4.	3	219	11.27789	Pyrophosphatase activity	*FliF, FliG, FliM*
5..	3	228	10.83271	Hydrolase activity, acting on acid anhydrides	*FliF, FliG, FliM*

**Fig. 5:**
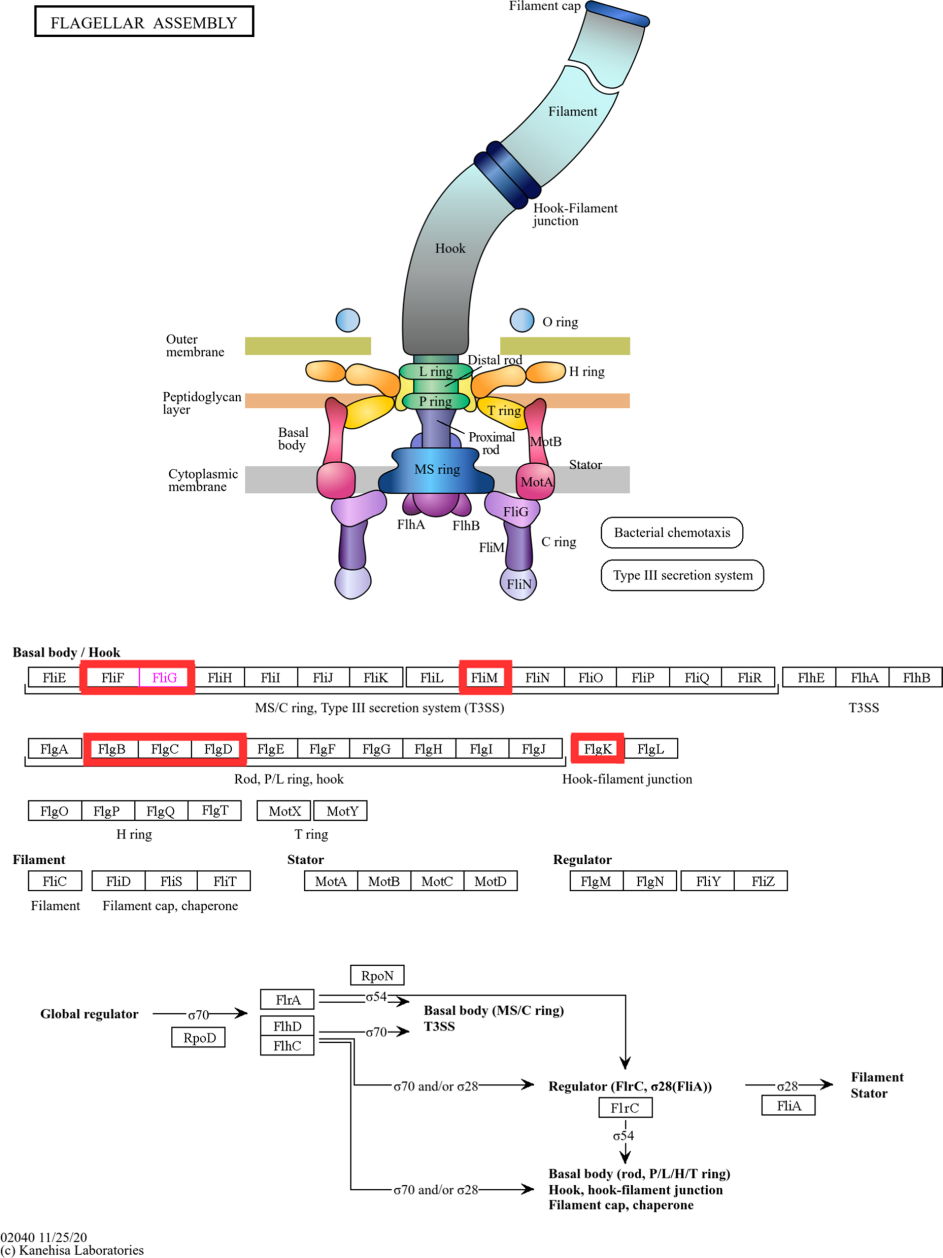
Schematic diagram of the flagellar assembly pathway (KEGG pathway). Hub genes are highlighted in RED

Enriched KEGG pathways were also analysed, as it provides valuable data for identified hub genes association in different pathways. The result is shown in Fig. 5. It is evident that the majority of the identified hub genes play an important role in the bacterial flagellar assembly pathway.

### Docking and MM-GBSA estimation

In our present study, a total of 17,967 phytochemicals were initially considered from the IMPPAT database. A series of drug-likeness filters, including the weighted Quantitative Estimate of Drug-likeness (QEDw > 0.7) and Lipinski’s Rule of Five (LO5), were applied to refine the dataset, ultimately yielding 401 compounds with favourable pharmacokinetic properties. Subsequently, these phytochemicals were subjected to XP docking. The docking score for the hub proteins in complex with the ligands ranged from − 7.71 to − 6.13 kcal/mol, indicating favourable binding interactions (Table 6). To assess the stability of the binding complex, the binding free energy was estimated using the Prime MM-GBSA approach. The binding free energies for the docked complexes vary between − 48.18 and − 35.58 kcal/mol (Dasmahapatra et al. 2022). The energy components showed that the total binding affinity was mainly driven by van der Waals and lipophilic contributions, which adds to the overall stability of protein–ligand interactions (Table 6). Collectively, the observations reinforce the virtual screening findings, demonstrating that the phytochemicals are capable of targeting flagellar-associated hub proteins crucial for bacterial motility and biofilm-driven resistance.

**Table 6:** Molecular interaction profiles of selected ligands with hub genes

S. No	Hub genes	Ligand	XP GScore (kcal/mol)	ΔG Bind (kcal/mol)	Covalent energy (kcal/mol)	Lipophilic energy (kcal/mol)	Van der Waals energy (kcal/mol)
1.	*FliM*	IMPHY006251	− 7.714	− 47.070	3.810	− 14.740	− 28.710
2.	*FlgD*	IMPHY003007	− 7.397	− 46.830	12.160	− 28.390	− 29.700
3.	*FlgB*	IMPHY010075	− 7.053	− 44.400	5.480	− 12.310	− 26.440
4.	*FliG*	IMPHY015946	− 7.017	− 41.550	1.230	− 14.590	− 34.850
5.	*FliF*	IMPHY000079	− 6.959	− 36.200	2.790	− 9.520	− 28.780
6.	*FlgC*	IMPHY000655	− 6.542	− 40.200	8.100	− 4.000	− 22.260
7.	*FlgK*	IMPHY010647	− 6.135	− 35.580	3.220	− 15.930	− 25.980

### Binding mode analysis

**Fig. 6:**
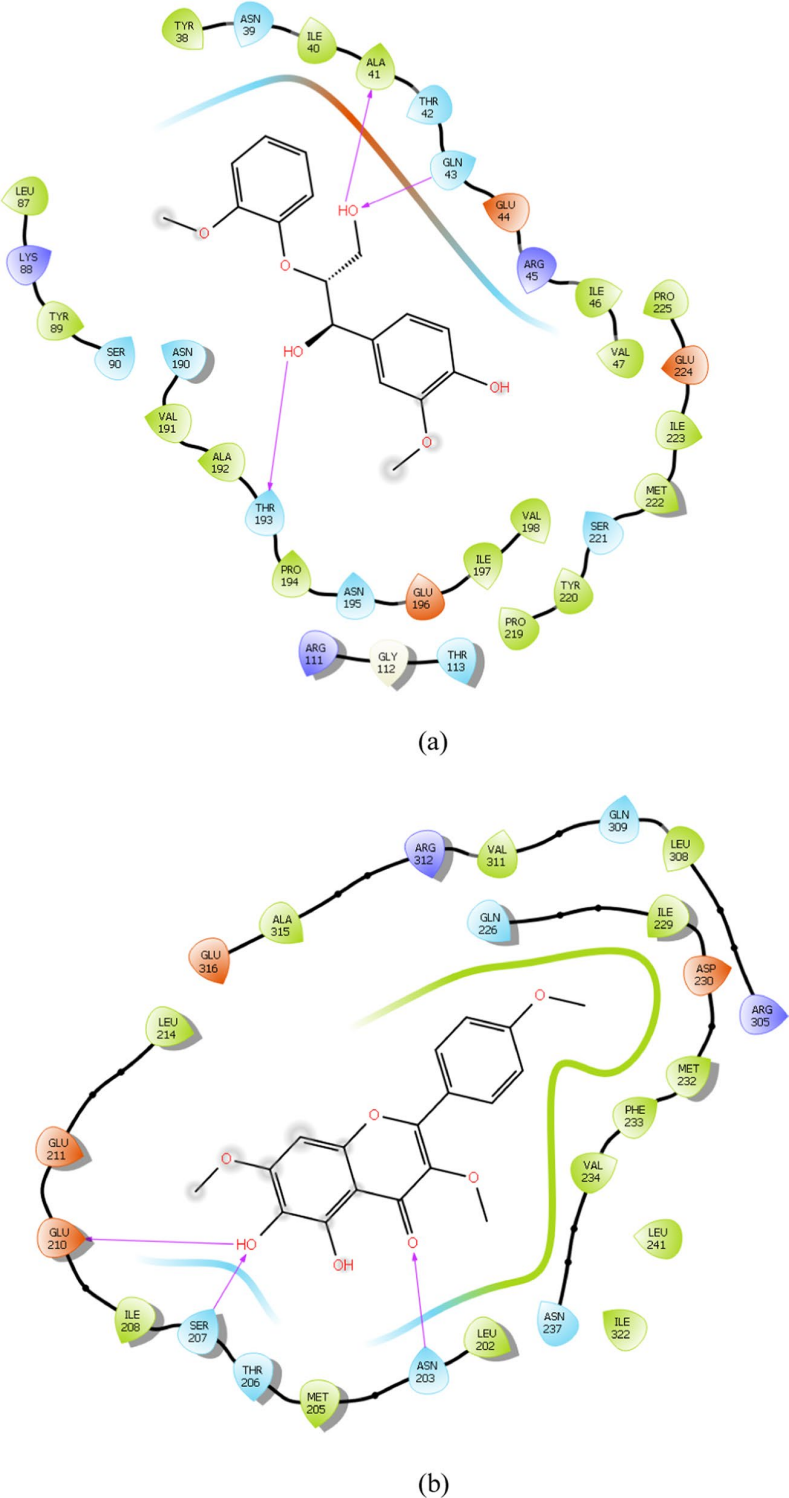

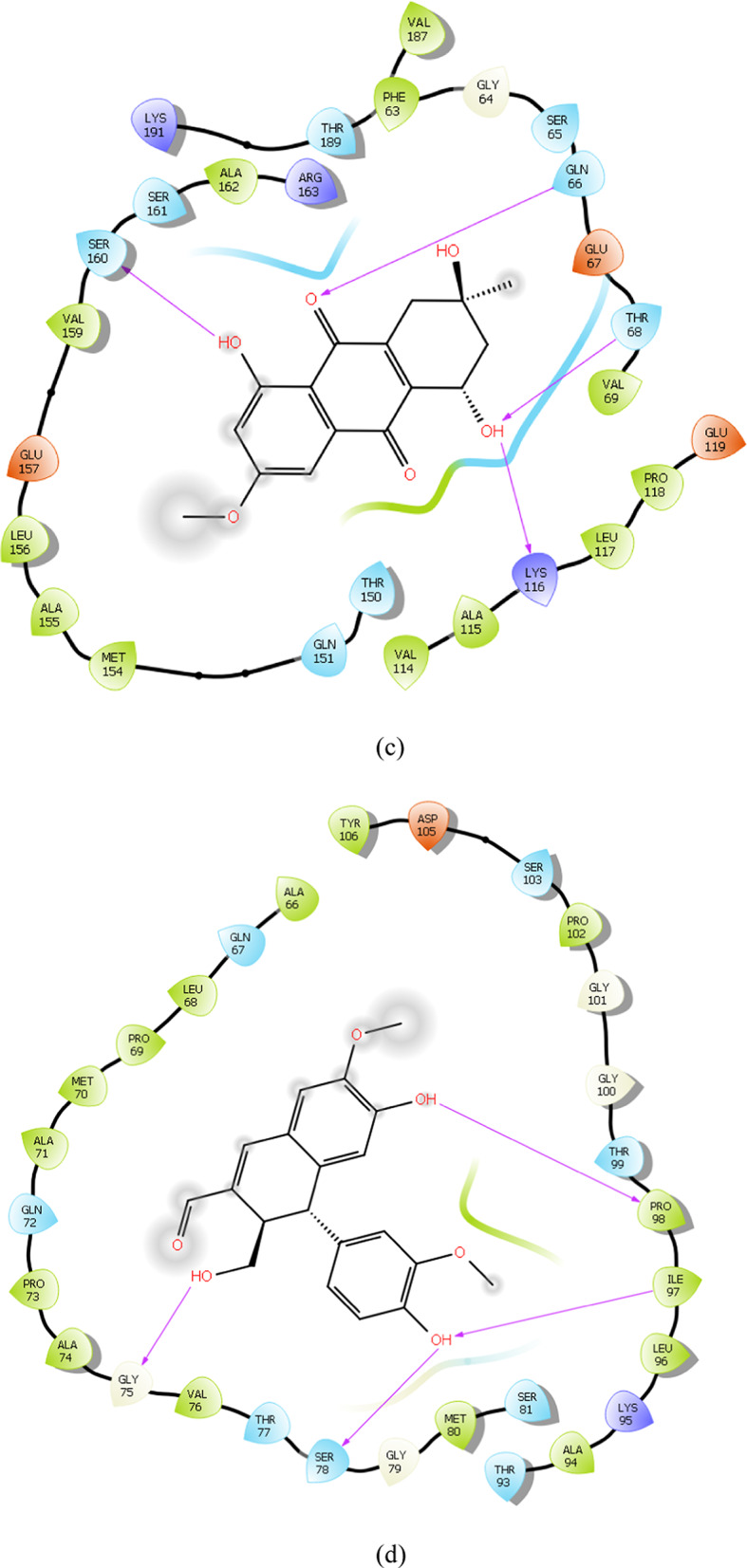

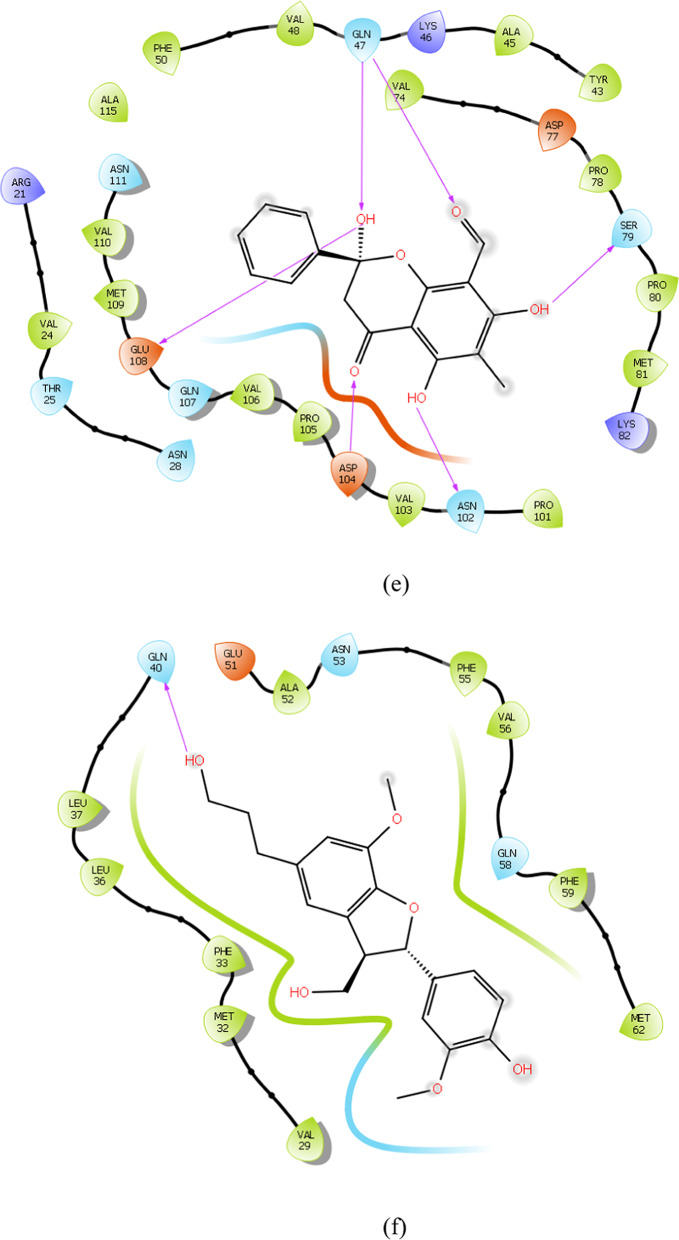

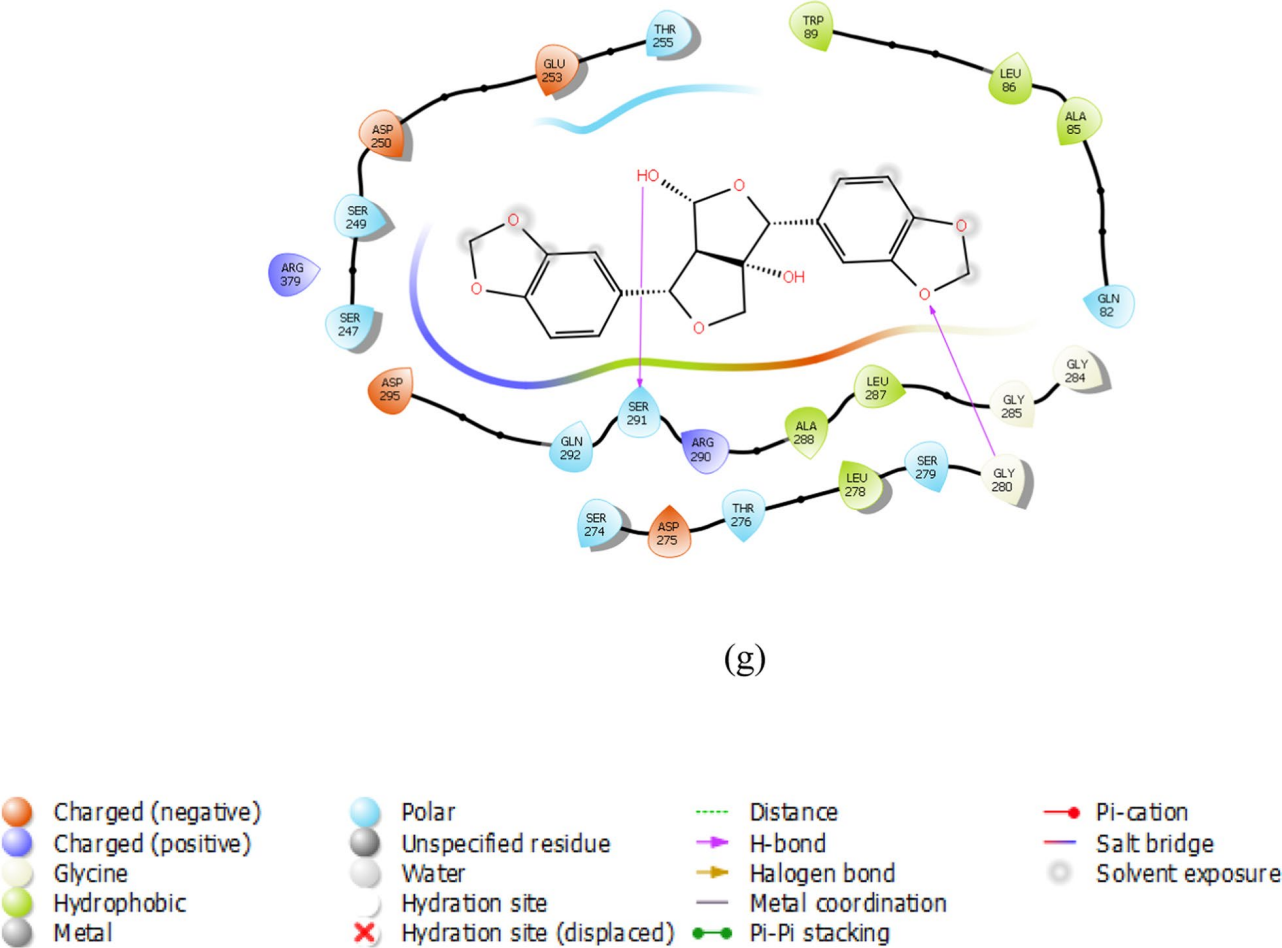
Binding mode of phytochemicals in the active site of the hub genes. **a** IMPHY06251 in *FliM*, **b** IMPHY015946 in *FliG*, **c** IMPHY000079 in *FliF*, **d** IMPHY010075 in *FlgB*, **e** IMPHY000655 in *FlgC*, **f** IMPHY003007 in *FlgD*, **g** IMPHY010647 in *FlgK*

To gain deeper insight into the binding mechanism of the reference and lead compounds, the binding conformations and key catalytic pocket residues of the target protein were analysed. Importantly, hydrogen bonds are key determinants of protein structural stability and compact folding (Pinto et al. 2023). The interaction analysis revealed that several phytochemicals formed stable complexes with the identified hub genes through multiple hydrogen bonding interactions. For instance, *FliM*–IMPHY006251 and *FliG*–IMPHY015946 each formed three hydrogen bonds with key residues Ala41, Gln43, Thr193 and Glu210, Ser207, Asn203 respectively Fig. 6a, b. *FliF*–IMPHY000079 established four hydrogen bonds with Ser160, Gln66, Thr68 and Lys116 (Fig. 6c). *FlgB*–IMPHY010075 displayed strong binding through four hydrogen bonds, involving Gly75, Ser78, Ile97, Pro98 (Fig. 6d). *FlgC*–IMPHY000655 established six hydrogen bonds with Glu108, Gln47, Asp104, Gln47, Ser79, Asn102 (Fig. 6e). *FlgD*–IMPHY003007 formed a single hydrogen bond with Gln40 (Fig. 6f). Additionally, *FlgK*–IMPHY010647 established two hydrogen bonds with Ser291, Gly280 (Fig. 6g). These consistent interactions with essential residues underscore the potential of the phytochemicals to disrupt bacterial motility and virulence.

## Discussion

The bacterial flagellum is a proteinaceous organelle made up of filament, hook and a basal body responsible for pathogenic invasion of the host, facilitating optimum site selection, colonization, maintenance of the infected site and post-infection dispersion (Chaban et al. 2015; Tan et al. 2024). As detailed in Fig. 5**,** the basal body comprises the structural elements such as L ring, MS ring, C ring, rod and export apparatus. These components are functionally integrated to drive flagellar rotation and torque generation. The L ring serves as a pivot to stabilize the rod's rotation, acting as the driving shaft where *FlgB, FlgC* and *FlgD* genes contribute to the rod's rotation. The MS ring, located on the inner membrane, consists of two overlapping rings, the M-ring and the S-ring, exclusively formed by numerous copies of the protein *FliF* (Mazzantini et al. 2024**)**. In the cytoplasmic C-terminus of the MS-ring, *FliF* interacts with the N-terminus of the C-ring component *FliG*, establishing a linkage between rotor components that facilitates flagellar rotation (Miethke et al. 2021; Kinoshita et al. 2018)**.** The *FliG* component comprises many subdomains. The C-terminal domain of *FliG (FliG*_*C*_), comprising the *FliG*_*CN*_ and *FliG*_*CC*_ subdomains, engages with the stator and facilitates torque generation and transfer. The central domain of *FliG (FliG*_*M*_) associates with *FliM*, which concurrently interacts with *FliN* (Tan et al. 2024). Therefore, torque generation and transfer facilitate the bacterial colonization which promotes biofilm formation and infection. Collectively, this intricate system facilitates the mechanism of chemotaxis. Chemotaxis is a process that fosters bacterial adherence and the formation of biofilm. This behaviour has been found in several ecological and cultural contexts among distinct bacterial species. Additionally, chemotaxis pathway governs excitation and adaptability to environmental cues, crucial for bacterial survival, metabolism and ecological interactions. In light of this, MS ring consists of the hub gene *FliF* which is crucial for the rotation of the bacterium. The hub gene *FliF* involves in the formation of flagella which is the critical step for the motility and virulence. The hub genes *FliG* and *FliM* help in the torque generation which aids the *B. cenocepacia* and *B. multivorans* bacterial colonization and biofilm formation which causes nosocomial infection in cystic fibrosis patients.

The flagellar hook protein, *FlgK* plays a crucial role in the formation and functionality of the bacterial flagella, which is vital for motility and colonization (Lv et al. 2024; Lu et al. 2023). This gene is located at the junction of filament and the hook which is essential for normal flagellar formation. Additionally, two types of bacterial motility are associated with the flagellum that are swarming and swimming. Swarming motility refers to the mass movement of moving bacteria from the inoculation site to the surrounding area on the surface of the medium, which relies on flagella. It provides an ecological advantage for bacteria by enabling clusters of bacteria to rapidly migrate to favourable environment, absorb and colonize clusters in host tissues (Pastora et al. 2024). Swimming motility is considered a significant virulence factor for certain bacteria, facilitating the rapid dissemination of pathogens from the primary infection site to various tissues, thereby leading to life-threatening infections. In this regard, research towards vaccine development has underscored the immunological significance of flagellar proteins *FliC* and *FlgL* in *Burkholderia pseudomallei* (Badten and Torres 2024)*.* Emerging evidence suggests considerable cross reactivity and sequence conservation with homologous genes in *Burkholderia cepacia* complex. *FliC* has shown significant protective immunogenicity in preclinical models of melioidosis and shows 84% sequence similarity with BCC. Therefore, these findings highlight the conserved characteristics of flagellar components within the *Burkholderia* genus. Moreover, identification of key hub genes reinforces the critical role of flagellar components in motility, pathogenicity and biofilm formation.

Our study demonstrates that these motility-associated hub genes are intricately connected to resistance phenotypes. For instance, Pinto et al. (2023) highlighted the flagellar genes *FliM* and *FliA* plays a pivotal role in virulence and resistance in *Stenotrophomonas maltophilia*. Findings indicate that the flagellum not only drives motility but also facilitates resistance associated biofilm formation and host persistence. Notably, targeting conserved pathway (flagellar assembly) disrupt colonization, biofilm formation and antibiotic tolerance mechanisms that particularly robust in the thick mucus environment of CF patients.

Furthermore, the inclusion of molecular docking and MM-GBSA binding energy analysis adds a crucial layer of structural validation to our *in-silico* findings. These methods provide robust evidence for the feasibility of targeting flagellar associated hub proteins using phytochemicals. Moreover, essentiality analysis confirmed that these hub genes are vital for bacterial survival, reinforcing their potential as therapeutic targets. The present research represents a detailed computational framework for identifying and prioritizing potential therapeutic targets in *Burkholderia*. Despite this, we comprehend that experimental validation is essential to confirm the functional roles and therapeutic significance of the identified hub genes. Nevertheless, the *in-silico* results provide a significant foundation to strengthen subsequent validation efforts, efficiently identifying essential targets for concentrated experimental exploration. To highlight, from the identified nodes, seven hub nodes play a crucial role in motility and colonization, which aid in biofilm formation and lead to AMR. Therefore, these crucial hub genes can be targeted for drug discovery or repurposing to overcome antimicrobial/antibacterial resistance.

## Conclusion

The discovery of key genes linked to antibiotic resistance in *B. cenocepacia* and *B. multivorans* marks a significant step forward in tackling the difficulties encountered by individuals with cystic fibrosis. We used algorithms like STRING and Cytoscape not only to disclose the genomic signature but also to identify the novel therapeutic targets for the resistance management. Importantly, analysis identified a significant connection between these hub genes and the flagellar assembly pathway, an essential component of bacterial motility, colonization and biofilm formation. Seven essential genes were recognized as vital elements in the facilitation of resistance mechanisms and further validation through molecular docking and MM-GBSA analysis confirmed strong and stable interactions between selected phytochemicals and the identified hub genes. Our study is the first to report the involvement of flagellar assembly pathway in mediating antimicrobial resistance and virulence in these pathogens. Ultimately, this research provides a robust foundation for addressing the global challenge of antibiotic resistance, especially to susceptible patient population. By unveiling genetic and molecular pathway we can develop precise, effective and sustainable strategies to combat chronic infections. Additionally, factors such as horizontal gene transfer, environmental influences and gene expression dynamics were not explored but are critical in shaping bacterial resistance and virulence. Future studies incorporating these factors along with in vitro and in vivo validation, will be crucial to fully understand the translational potential of these findings. Indeed, the results open avenues for focused strategies, providing optimism for improved treatment of chronic infections in individuals with cystic fibrosis. Overall, achieving these objectives offers a promising direction for precision-targeted strategies against antimicrobial resistance in cystic fibrosis, paving the way for improved clinical outcomes and sustainable therapeutic interventions.

## Data Availability

All data analysed during this study are publicly available and are included in this article. The datasets analysed during the current study are available in the NCBI repository (https://www.ncbi.nlm.nih.gov/).

## Abbreviations

AMR: Antimicrobial resistance
CF: Cystic fibrosis
VF: Virulent factors
MCODE: Molecular complex detection
BCC: *Burkholderia* *cepacia* Complex
BPC: *Burkholderia* *pseudomallei*
CFTR: Cystic fibrosis transmembrane conductance regulator gene
MDR: Multidrug resistant
NCBI: National centre for biotechnology information
CARD: Comprehensive antibiotic resistance database
VFDB: Virulence factor database
PPI: Protein–protein interaction
STRING: Search tool for the retrieval of interacting genes
DEG: Database of Essential Genes
IMPPAT: Indian Medicinal Plants, Phytochemistry and Therapeutics
QEDw: Quantitative Estimate of Drug-likeness
XP: Extra Precision
MM-GBSA: Molecular Mechanics-Generalized Born Surface Area
FDR: False discovery rate
BP: Biological process
CC: Cellular component
MF: Molecular function
